# Fecal microbial transplantation limits neural injury severity and functional deficits in a pediatric piglet traumatic brain injury model

**DOI:** 10.3389/fnins.2023.1249539

**Published:** 2023-09-28

**Authors:** Madison M. Fagan, Christina B. Welch, Kelly M. Scheulin, Sydney E. Sneed, Julie H. Jeon, Morgane E. Golan, Savannah R. Cheek, Deborah A. Barany, Georg Oeltzschner, Todd R. Callaway, Qun Zhao, Hea Jin Park, Jeferson M. Lourenco, Kylee J. Duberstein, Franklin D. West

**Affiliations:** ^1^Regenerative Bioscience Center, University of Georgia, Athens, GA, United States; ^2^Biomedical and Health Sciences Institute, University of Georgia, Athens, GA, United States; ^3^Department of Animal and Dairy Science, College of Agricultural and Environmental Sciences, University of Georgia, Athens, GA, United States; ^4^Department of Nutritional Sciences, College of Family and Consumer Sciences, University of Georgia, Athens, GA, United States; ^5^Department of Kinesiology, College of Education, University of Georgia, Athens, GA, United States; ^6^Russell H. Morgan Department of Radiology and Radiological Science, The Johns Hopkins University School of Medicine, Baltimore, MD, United States; ^7^Department of Physics and Astronomy, Franklin College of Arts and Sciences, University of Georgia, Athens, GA, United States

**Keywords:** traumatic brain injury, porcine (pig) model, fecal matter transfer, MRI, microbiome gut-brain axis, behavior analysis, gait analysis

## Abstract

Pediatric traumatic brain injury (TBI) is a leading cause of death and disability in children. Due to bidirectional communication between the brain and gut microbial population, introduction of key gut bacteria may mitigate critical TBI-induced secondary injury cascades, thus lessening neural damage and improving functional outcomes. The objective of this study was to determine the efficacy of a daily fecal microbial transplant (FMT) to alleviate neural injury severity, prevent gut dysbiosis, and improve functional recovery post TBI in a translational pediatric piglet model. Male piglets at 4-weeks of age were randomly assigned to Sham + saline, TBI + saline, or TBI + FMT treatment groups. A moderate/severe TBI was induced by controlled cortical impact and Sham pigs underwent craniectomy surgery only. FMT or saline were administered by oral gavage daily for 7 days. MRI was performed 1 day (1D) and 7 days (7D) post TBI. Fecal and cecal samples were collected for 16S rRNA gene sequencing. Ipsilateral brain and ileum tissue samples were collected for histological assessment. Gait and behavior testing were conducted at multiple timepoints. MRI showed that FMT treated animals demonstrated decreased lesion volume and hemorrhage volume at 7D post TBI as compared to 1D post TBI. Histological analysis revealed improved neuron and oligodendrocyte survival and restored ileum tissue morphology at 7D post TBI in FMT treated animals. Microbiome analysis indicated decreased dysbiosis in FMT treated animals with an increase in multiple probiotic *Lactobacilli* species, associated with anti-inflammatory therapeutic effects, in the cecum of the FMT treated animals, while non-treated TBI animals showed an increase in pathogenic bacteria, associated with inflammation and disease such in feces. FMT mediated enhanced cellular and tissue recovery resulted in improved motor function including stride and step length and voluntary motor activity in FMT treated animals. Here we report for the first time in a highly translatable pediatric piglet TBI model, the potential of FMT treatment to significantly limit cellular and tissue damage leading to improved functional outcomes following a TBI.

## Introduction

Pediatric traumatic brain injury (TBI) is a substantial contributor to death and disability worldwide. Each year in the United States alone there are over 2.8 million TBI hospital visits of which over 850,000 are children (CDC Injury Center, 2022). TBI results in brain lesioning, edema, hemorrhage, cerebral blood flow disruption, and inflammation that can have widespread effects on the body including gut dysbiosis (Feighery et al., 2008; Sundman et al., 2017) and loss of gastrointestinal tract (GIT) integrity (Hang et al., 2003). In children, TBI can interrupt and irrevocably alter ongoing neural development, thus leading to lifelong deficits in learning (Prasad et al., 2017), memory (Prasad et al., 2017), motor function (Beretta et al., 2009), and behavior (Li and Liu, 2013). As such, TBI is considered a chronic disease as opposed to a static injury (Masel and DeWitt, 2010). Although TBI is a worldwide epidemic, there is no FDA-approved treatment. Therefore, there is a significant need for the development of novel and innovative TBI therapies that will lead to improved patient recovery. Recent studies in rodent TBI models have demonstrated that manipulating the GIT microbiome through fecal microbiota transplantation (FMT) (Du et al., 2021; Davis et al., 2022) or introduction of specific probiotic species, *Lactobacillus acidophilus* (Sun et al., 2015; Ma et al., 2019) or *Clostridium butyricum*, (Li et al., 2018) may lead to reduced intracerebral brain damage and improved cognitive and motor function outcomes, making the GIT microbiome a novel TBI therapeutic target.

A number of recent studies have discovered significant connections between the gut microbiome and brain health (Tremlett et al., 2017). The gut microbiome consists of over a trillion microorganisms that primarily inhabit the lower portion of the GIT system. Connected through the microbiota-gut-brain-axis (MGBA), bi-directional communication exists in which the gut microbiome communicates to the brain through the immune system, enteric nervous system, and hypothalamic-pituitary-adrenal (HPA) axis using complex signaling molecules (Rice et al., 2019). Indeed, TBI can result in changes to the gut microbiome which have been detected as early as 2 h after experimental TBI (Crapser et al., 2016; Fung et al., 2017; Nicholson et al., 2019; Jeon et al., 2020). These changes often include an increase in opportunistic pathogenic bacteria commonly associated with disease and a corresponding decrease in healthy, commensal bacteria resulting in a population imbalance, termed dysbiosis. GIT dysbiosis leads to the activation of GIT inflammatory cells, breakdown of the intestinal wall, and leakage of intestinal contents and bacteria into the circulatory system which results in systemic inflammation that further exacerbates TBI induced inflammation in the brain. Additionally, the vagus nerve and HPA axis are activated in a positive feedback loop which perpetuates inflammation in both the brain and the GIT following TBI (Thayer and Sternberg, 2009). Therefore, a microbial based therapy may act on each arm of the MGBA to reduce the TBI-induced secondary injury cascade and improve outcomes.

As interest in the gut microbiome-health connection increases, evidence has grown that introduction of key gut bacteria may contribute to the treatment of numerous central nervous system (CNS) pathologies such as Alzheimer’s Disease, Parkinson’s Disease, and TBI (Vendrik et al., 2020). Recent studies have demonstrated that FMT may be a potent microbiome altering therapeutic option for TBI (Du et al., 2021; Davis et al., 2022). FMT is the ingestion of a healthy bacterial population isolated from donor stool with the goal of replenishing a dysbiotic gut microbiome. FMT studies in controlled cortical impact (CCI) TBI mouse models showed that TBI induced significant gut dysbiosis, including changes in microbial population alpha- and beta-diversity, with increasing levels of dysbiosis correlating with increased brain lesion volumes (Li et al., 2018). FMT treatment restored gut microbial populations back to a pre-injury state (Du et al., 2021; Davis et al., 2022). FMT-induced restoration of the microbiome was associated with decreased oxidative stress, cortical volume loss, and attenuated white matter damage in the brain (Du et al., 2021; Davis et al., 2022). FMT-treated animals also showed reduced neurological deficits as measured by improved modified neurological severity scores, decreased anxiety-like behavior as determined by open field testing, and improved spatial memory and learning as determined by Morris Water Maze testing (Du et al., 2021; Davis et al., 2022). These studies demonstrate that FMT is a promising therapeutic that can limit TBI pathogenesis and lead to improved recovery in rodent models.

Despite highly compelling FMT TBI therapeutic data in rodent systems, significant differences exist in human and rodent anatomy and physiology that may decrease the translational potential of FMT into human patients, particularly in the pediatric population. Porcine models are increasingly utilized in neural injury studies due to key similarities in human and pig anatomy and physiology (Kinder et al., 2019c; Kaiser and West, 2020). In fact, humans and pigs share remarkably similar neuroanatomical architecture, including both possessing gyrencephalic brains, similar white matter composition (>50%), and cerebral vasculature (Ahmad et al., 2015; Kaiser and West, 2020). Evaluating the effects of TBI in a gyrencephalic brain is critically important as gyrification significantly influences the movement of the brain in the skull during TBI, including maximum mechanical stress in tissues, with considerably more brain deformation occurring and deeper transmission of forces relative to a lissenciphalic brain like those found in typical rodent models (Vink, 2018). The human brain is approximately 650 times the size of the average rodent brain, while only 7.5 times larger than the pig brain- a size comparable to non-human primate models (Lind et al., 2007). Brain size is an important factor when evaluating TBI effects on brain pathophysiology as smaller rodent brains can tolerate much greater angular acceleration forces than animals with larger brains as shearing forces and inertial loading are related to brain mass (Vink, 2018). Moreover, the piglet brain shows a more similar time course in neural development, maturation, and myelination to human adolescents as compared to the rodent model (Flynn, 1984; Conrad et al., 2012). Humans and pigs also share key GIT features including similarities in body size resulting in more relatively proportional intestinal size, length, and transit time as opposed to rodents (Heinritz et al., 2013). Pigs and humans share more similar digestion strategies as opposed to rodents. For example, fermentation occurs primarily in the colon of pigs and humans versus cecal fermentation in rodents (Miller and Ullrey, 1987). Studies have also demonstrated similarities in the gut bacterial populations in humans and pigs when compared to rodents (Lamendella et al., 2011). Both adult human and pig GIT microbial populations are dominated by Firmicutes and Bacteroidetes and show similar levels of microbial diversity (Lamendella et al., 2011). Furthermore, >95% of functional genes in the human gut metagenome are present in the pig, thus demonstrating significant overlap between the functional capacity of pig and human microbiomes and further supporting the use of porcine preclinical microbial models whereas less microbial functional gene overlap is observed between humans and rodents (Xiao et al., 2016). Finally, the pediatric piglet TBI model has also demonstrated relevant functional deficits after TBI including loss of motor coordination and behavioral changes (Baker et al., 2019; Kinder et al., 2019a,b; Wang et al., 2021). Taken together, these key similarities in human and pig brain, GIT, microbiome, and functional outcomes enable the pig to be an invaluable tool in preclinical neural injury research, specifically in modeling gut mediated therapies.

The objective of this study was to determine the efficacy of a daily FMT to alleviate neural injury severity, reverse GIT dysbiosis, and improve functional recovery post TBI in a translational pediatric porcine model. In this study, we demonstrate that FMT treatment leads to a significant reduction in lesion volume, midline shift, hemorrhage, and edema in our piglet TBI model. Furthermore, we demonstrate that FMT treatment prevents an acute increase in potentially harmful fecal microbial species and promotes an increase in cecal therapeutic probiotic species post-TBI. These FMT-mediated improvements in injury severity and gut dysbiosis led to enhanced recovery in motor function and behavioral outcomes in TBI piglets.

## Materials and methods

### Animals and housing

Four-week-old castrated male crossbreed piglets (*N* = 18) were used as experimental animals in this study due to animal availability. Previous research from our lab indicated that crossbreed animals showed similar brain pathophysiology and motor outcomes following TBI as Yucatan biomedical pigs (Sneed et al., 2020). Additional castrated male piglets (*N* = 9) were kept from each litter as healthy fecal matter donor pigs. All piglets were born from the same genetic lineage and maintained in an environmentally controlled room to minimize microbiome variability. This study was performed in accordance with the National Institutes of Health (NIH) Guide for the Care and Use of Laboratory Animals guideline. All procedures were reviewed and approved by the University of Georgia Institutional Animal Care and Use Committee (Animal Use Protocol A2019 07-007-Y1-A9). Pigs were housed in Public Health Service (PHS) and Association for Assessment and Accreditation of Laboratory Animal Care (AAALAC) approved facility with a 12-h light/dark cycle maintained at room temperature (27°C). Pigs were provided free access to water and fed standard pig starter I diets. Additionally, all pigs received daily enrichment through human contact and toys.

### Study design

A total of 18 animals were enrolled in this randomized single blinded study. To minimize day effects, this study was conducted in a randomized block design in which animals were randomly assigned to one of three treatment groups (Sham craniectomy + saline, TBI + saline TBI + FMT). Treatments were administered in an unblinded fashion; however, all data was anonymized and underwent blinded analysis. Beginning the day after birth, piglets were socialized and acclimated to human interaction in order to reduce handling stress during future procedures. Starting at 2 weeks of age, piglets were individually habituated to the gait track and underwent gait training 5 days per week for 2 weeks. After weaning at approximately 4 weeks of age, pre-injury gait and behavior data were collected, and pigs were randomly assigned to either TBI + FMT (FMT; *n* = 6), TBI + saline (TBI; *n* = 6) or craniectomy + saline (Sham; *n* = 6) experimental groups. FMT or saline treatments were administered 2-h post TBI or Sham surgery, and once a day thereafter. MRI collection occurred 1 and 7 days post-surgery. Gait, behavior, and fecal matter collections took place 1, 3, and 7 days post-surgery. All experimental animals were sacrificed 7 days post-surgery for tissue analysis and cecal collection.

### Controlled cortical impact

Approximately 12-h prior to surgery a transdermal fentanyl patch (12 mcg/h; Covetrus) was applied to each pig for pain management. Pre-induction analgesia and sedation for TBI or Sham surgery was achieved using xylazine (2 mg/kg IM; VetOne) and midazolam (0.2 mg/kg IM; Heritage). Prophylactic lidocaine (0.5 mL 2% lidocaine VetOne) was administered topically to laryngeal folds, and propofol (0.5 mL to effect IV; Zoetis) to facilitate intubation. Anesthesia was maintained with isoflurane (1.0–2.0% Abbott Laboratories) in oxygen. Vitals including heart rate, respiration, and temperature were continuously monitored throughout surgery and maintained within normal parameters.

Using aseptic sterile technique, 4 cm left-sided incision was made at the top of the cranium to expose underlying skull. Bupivacaine (0.5% bupivacaine, 2 mg/kg; Pfizer) was applied as a periosteal block for desensitization of the area. A 20 mm craniectomy was performed at the left anterior junction of the coronal and sagittal sutures, exposing the underlying dura of the motor cortex. Each pig was then secured in in a controlled cortical impact device (University of Georgia Instrument Design and Fabrication Shop, Athens, GA, USA). A 15 mm blunt impactor tip was centered over the exposed dura to induce a motor cortex TBI with the following parameters: 4 m/s velocity, 9 mm depth of depression, 400 ms dwell time. Studies conducted previously by our lab determined these parameters generate a moderate-severe TBI (Baker et al., 2019; Kinder et al., 2019a). Sham animals received craniectomy only with no TBI. After TBI or Sham induction, the injury site was flushed with sterile saline. A second dose of Bupivacaine (0.5% bupivacaine, 2 mg/kg; Pfizer) was administered as an incisional block prior to closing the tissue with surgical sutures.

Post-operatively, pigs were monitored continuously until extubated. Once returned to their pen, pigs were monitored every 15 min until vitals returned to normal, then every 4 h for 24-h, and twice daily thereafter.

### Gut microbial transplant

#### Collection

Healthy male piglets were kept from each litter as sex- and age-matched fecal matter donor pigs. Fecal matter was collected once per day beginning on the day of TBI induction, through day 6 of the study for the administration of fresh solution each day. For collection, a trained handler restrained each donor pig as a second trained handler cleaned the rectum with a 70% ethanol wipe. A sterile swap was used to stimulate the rectum. Fecal matter was collected into a sterile 50 mL conical tube and immediately placed on ice until all collections were complete and transported for processing.

#### Processing

All processing occurred in an anaerobic chamber. Fecal matter from each donor pig was combined and pooled into one sample to obtain a homogenous mixture to ensure all FMT treated piglets received the same treatments. Pooled fecal matter was then weighed and placed in a commercial blender (Oster Classic Series). Sterile saline was added to the blender based on weight to result in a 50 g fecal matter per 250 mL sterile saline ratio (Ponte et al., 2015), and blended on low for 30 s until a smooth texture was achieved. The mixture was then filtered through sterile gauze three times to ensure removal of particulate matter. Approximately 30 mL of microbial transplant material was placed into sterile syringes and immediately administered to FMT piglets.

#### Administration

Piglets were placed in individual pens for treatment administration in which 25 mL of the prepared 30 mL of microbial transplant material or saline were administered to FMT or saline piglets, respectively. Treatments were slowly dispensed orally via syringe feeding. Treatments were administered at 2 h post TBI or craniectomy, and once a day thereafter. All piglets were monitored for approximately 15 min after treatment to ensure no adverse effects (vomiting, diarrhea) occurred. However, no adverse events were noted for the duration of the study.

### Magnetic resonance imaging (MRI) acquisition and analysis

Magnetic resonance imaging was preformed 1 and 7 days post TBI using a General Electric 32-channel fixed-site Discovery MR750 3.0 Tesla MRI magnet and an 8-channel knee coil placed over the head. All pigs were sedated as previously described for craniectomy surgery, with the addition of ketamine (4 mg/kg IM; Henry Schein) at the 7 day scan as fentanyl was no longer in use. Pigs were placed in supine recumbency, and mild anesthesia was maintained via inhalation isoflurane (1.5%; Abbott Laboratories) in oxygen. Vitals were monitored for the duration of the scan, and until pigs recovered and were ambulatory. Multiplanar MRI sequences were acquired including Fast Spin Echo T2-Weighted (FSE T2W), susceptibility-weighted angiography (SWAN), diffusion weighted imaging (DWI), diffusion Tensor Imaging (DTI), and magnetic resonance spectroscopy (MRS). Sequence analysis was conducted using Osirix software (Version 12.5.2) default thresholds and Fiji ImageJ software (Version 2.0.0).

Lesion volume and midline shift were calculated from coronal FSE T2W sequences using Osirix software. To determine lesion volume, hypointense and hyperintense areas in the ipsilateral hemisphere were manually traced. Computer-generated lesion volumes were automatically calculated and reported. Similarly, each slice of the ipsilateral hemisphere was manually traced to calculate the percentage of which the lesion occupied (lesion volume divided by ipsilateral hemisphere volume). The midline shift (MLS) was measured at the falx cerebri and septum pellucidum (Yang et al., 2018). Specifically, the distance from each structure to the lateral border of the ipsilateral and contralateral hemisphere was measured. The MLS was then calculated using the formula: (total diameter/2)–contralateral diameter (Wang et al., 2021). Intracranial hemorrhage volume was measured with Osirix from coronal SWAN sequences. Similar to lesion volume, hypointense areas were manually outlined on each ipsilateral slice and automated computer-generated volumes produced. Apparent diffusion coefficient (ADC) maps were generated from DWI sequences and utilized within ImageJ software to measure changes in water diffusivity, corresponding to cytotoxic (hypointense regions) and vasogenic (hyperintense regions) edema. Specifically, each hemisphere was manually defined. Using the contralateral hemisphere as an internal control, the percent of abnormal (low or high, respectively) signal intensity was automatically calculated for each slice and averaged for a final value.

Proton magnetic resonance spectroscopy (1H-MRS) and unsuppressed water reference data were acquired in two voxels (16 × 20 × 25 mm^3^) using a standard point-resolved single voxel spectroscopy (PRESS) sequence (TR/TE = 2,000/35 ms; 8 averages; 5 kHz bandwidth with 4,096 data points; scan time 2:56 min per voxel). The first voxel was placed in the ipsilateral hemisphere to cover the lesioned area, and the second voxel was placed symmetrically in the contralateral hemisphere, with adjustments to avoid the skull. The voxel size was selected to best fit within the piglet brain and to maximize signal-to-noise and spectrum quality. Pre-scan shimming was performed to achieve a suitable line width (FWHM < 13 Hz). All MRS data were analyzed offline in Osprey software (version 1.1.0) (Oeltzschner et al., 2020) using the standard processing pipeline. Briefly, preprocessing steps include spectral frequency- and phase correction, spectral alignment and averaging, Fourier transformation, residual water removal, and linear baseline correction. The processed averaged spectra were then fitted using LCModel’s default linear-combination algorithms (Provencher, 1993) and baseline knot spacing of 0.40 ppm, as implemented in Osprey. Levels of total N-acetyl aspartate (NAA) and N-acetyl aspartyl glutamate (NAAG) (NAA + NAAG), and creatine and phosphocreatine (Cr + PCr) were estimated using water as a reference. For these peaks, model fit quality was high, as indicated by low Cramer-Rao Lower Bounds (<15% for all metabolites on all spectra). We also estimated levels of Cr and PCr separately, Lactate (Lac), and glutamate (Glu) as exploratory analyses; however, the quality of the model fits for these metabolites were often lower (Cramer-Rao Lower Bound exceeding > 15%). Though metabolite levels are commonly expressed as a ratio relative to total creatine (Cr + PCr), it is not used here as creatine levels can be variable, especially after brain injury (Ross et al., 1998; Rae, 2014).

### Histology

All pigs were euthanized following the 7 day post TBI MRI via euthanasia solution (1 mL/10 lbs IV). Immediately after euthanasia, pigs’ brains were removed and coronally sectioned at the level of the optic chiasm. The rostral portion of the brain was placed in 10% buffered formalin for immunohistochemical analysis. After fixation, the brain was further sectioned in which two serial ipsilateral slices at the level of the lesion which were routinely processed, embedded in paraffin, and stained with antibodies specific to NeuN (1:500), glial fibrillary acidic protein (GFAP; 1:4,000), Olig2 (1:400), Iba1 (1:8,000), and doublecortin (DCX; 1:2,000). Additionally, 5 cm of the distal ileum were dissected, cleaned, and placed in 10% buffered formalin. After fixation, three serial slices of the distal end of the ileal segment were routinely processed, embedded in paraffin wax, and stained with hematoxylin/eosin (H&E) for morphological analysis.

All slices of both gut and brain tissue were imaged using a Cytation 5 Imaging Multi-mode reader (BioTek, Vermont) in montage mode at 4 × magnification. Using Gen5 Software (Biotek, Vermont), images were digitally stitched together to create a single image. Quantification of brain cells was performed using ImageJ software by counting cell numbers (NeuN + neurons) or calculating the number of stained pixels per brain slice as indicated by percent positive staining (Olig2 + oligodendrocytes, Iba1 + microglia, GFAP + astrocytes, and DCX + neuroblasts). Villus height (measured from the top of the villus to the villus-crypt junction), and crypt depth (measured from the villus crypt junction to the base of the crypt) were measured using Gen5 Software. Measurements were conducted on 15 well-oriented, intact villi and their associated crypt for each pig.

### Microbiome analysis

#### Fecal collection and storage

Fecal samples were collected pre, 1, 3, and 7 days post TBI. Fecal samples were collected using aseptic equipment directly from the rectum of piglets. Piglets were restrained by a trained handler during collection. Prior to collection, the piglet’s rectum was cleaned using 70% ethanol wipes to avoid contamination. Sterile swabs were utilized to stimulate defecation, and feces was collected and stored in sterile 50 mL conical tubes. Samples were immediately frozen and stored at −80°C until further analysis.

#### Cecal content collection and storage

On day 7 after euthanized, the abdominal cavity of all piglets was dissected using sterilized tools. The cecum of all piglets was located by identifying the ileocecal junction. Using sterilized scissors, the cecum was opened and cecal contents were collected directly into sterile 50 mL conical tubes. Samples were immediately frozen and stored at −80°C until further analysis.

#### DNA extraction, sequencing, and analysis

Deoxyribonucleic acid extraction was performed on fecal and cecal samples following a previously described protocol with slight modifications (Welch et al., 2020). Briefly, 350 mg of sample was placed in a 2 mL Lysing Matrix E tube (MP Biomedicals LLC, Irvine, CA, USA), and homogenized using a QIAGEN vortex adapter (QIAGEN, Venlo, Netherlands) to disrupt the cells. Enzymatic inhibition was achieved by using InhibitEX Buffer (QIAGEN, Venlo, Netherlands), and DNA elution and purification was carried out using a spin column and a series of specialized buffers according to manufacturer’s specifications (QIAamp Fast DNA Stool Mini Kit; QIAGEN, Venlo, Netherlands). Determination of DNA concentration and purity in the resulting eluate was performed spectrophotometrically using the Synergy LX Multi-Mode Microplate Reader in conjunction with the Take3 Micro-Volume Plate (BioTek Instruments Inc., Winooski, VT, USA). Samples with a minimum volume of 100 μL and 10 ng/μL of DNA were stored at −20°C.

Following DNA extraction, samples underwent library preparation and 16S ribosomal ribonucleic acid (rRNA) gene sequencing. The library preparation step included polymerase chain reaction (PCR) replications using the forward: S-D-Bact-0341-b-S-17 (5′-CCTACGGGNGGCWGCAG-3′) and reverse: S-D-Bact-0785-a-A-21 (5′-GACTACHVGGGTATCTAATCC-3′) primer pairs (Klindworth et al., 2013), followed by a PCR clean-up using AMPure XP beads (Beckman Coulter Life Sciences, Indianapolis, IN, USA). A second PCR step was then carried out to attach Illumina’s indices and sequencing adapters (Nextera XT Index Kit; Illumina Inc., San Diego, CA, USA), followed by another PCR clean-up step using AMPure XP beads. Following the final clean up, the library was quantified using quantitative PCR (qPCR), and the nucleotides were sequenced using an Illumina MiSeq instrument and a MiSeq v3 reagent kit (Illumina Inc., San Diego, CA, USA). A well-characterized bacteriophage PhiX genome (PhiX Control v3 Library; Illumina Inc., San Diego, CA, USA) was used as a control for the sequencing runs.

Sequencing data was demultiplexed and converted to FASTQ files. Pair-end reads were converted into QIIME 2 artifacts (Bolyen et al., 2019) for further processing as described previously (Akerele et al., 2022). Sequences were denoised, dereplicated, and chimera-filtered using the DADA2 plugin (Callahan et al., 2016). A pre-trained Naïve Bayes classifier (Pedregosa et al., 2011) was utilized to assign taxonomies using the SILVA 138 SSU database (Quast et al., 2013). Even sequencing depth was set to 28,145 sequences per sample for computation of alpha and beta diversity indexes.

### Gait analysis

Pigs underwent gait analysis collection on three separate days pre-TBI, as well as 1, 3, and 7 days post-injury. Data were recorded using a GAITFour electronic pressure sensitive mat (CIR Systems, Inc.) that is 7.01 × 0.85 meters in size. The mat contains a 6.10 × 0.61 meter active area will a total of 23,040 sensors. Before weaning, pigs were habituated to the gait mat in small groups. Approximately 2 weeks before TBI, pigs were individually trained to travel across the mat at a consistent two beat trotting gait. After training, pigs were asked to travel across the mat until 5 consistent runs were collected, or for a maximum of 15 min. Individual runs with less than 10% variability in stride cycle velocity were kept for potential analysis. Runs were then selected for analysis based on the individual stride cycle velocity falling within 20% of the mean stride cycle velocity on each trial day. Trials were normalized to pre-TBI values to account for high levels of inter-pig variability.

Gait was semi-automatically analyzed using GAITFour Software to provide quantitative measurements for all limbs. Parameters analyzed include stride length (the distance between successive ground contact of the same hoof) and step length (the distance between corresponding successive points of contact of opposing hooves) for all four limbs of each animal.

### Open field testing

Pigs underwent open field behavior testing as an additional measure of functional outcomes pre-TBI as well as 1, 3, and 7 days post. All tests occurred in a 2.7 × 2.7 meter arena. Trials lasted 10 min and were recorded using EthoVision video tracking software (Noldus Systems) to obtain objective and quantifiable measures of behavioral characteristics such as velocity, distance traveled, and movement. Exploratory behaviors (sniffing) were manually tracked and coded by trained personnel.

### Statistical analysis

All quantitative data were analyzed using Minitab^®^ Statistical Software (version 21.1.1.0, Pennsylvania). For MRI, fecal microbiome, and behavior data, a mixed effects model was used in which treatment nested in pig was a random factor and time, treatment, and treatment by time were fixed effects. Differences were measured with *post-hoc* Tukey–Kramer pairwise comparisons. For cecal microbiome and histological measurements, statistical significance between groups was determined with one-way ANOVA and *post-hoc* Tukey–Kramer pairwise comparisons. Due to high levels of individual gait variability, significance between groups was measured at each timepoint using one-way ANOVA with *post-hoc* Fisher pairwise comparison. All results are reported as mean ± standard error of the mean (M ± SEM) using model adjusted values; however, figures are presented using the raw unadjusted values. *P*-values < 0.05 were considered significantly different, and *p*-values between 0.05 and 0.10 were considered trending.

## Results

### FMT decreases lesion volume and midline shift

**FIGURE 1:**
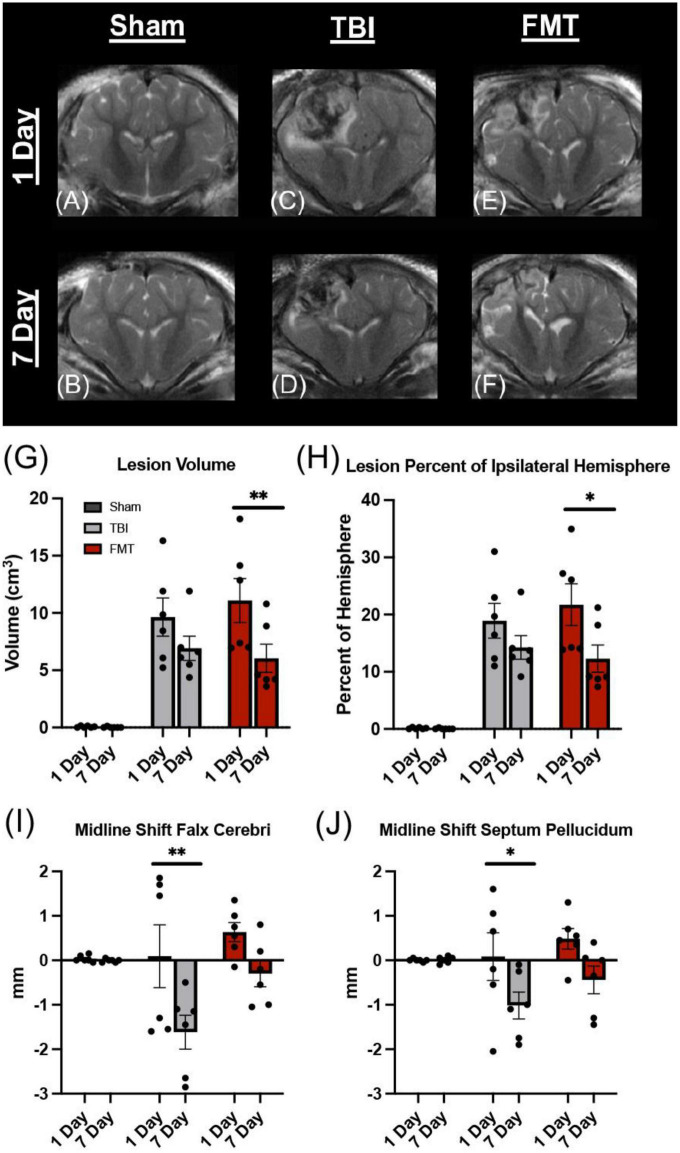
FMT significantly reduced lesion volume and MLS following TBI. Coronal T2W MRI scans revealed minimal to no lesion present in Sham animals at 1 and 7 days **(A,B)**. Hyperintense and hypointense ipsilateral lesioning characteristic of heterogenous TBI were present at 1 and 7 days in TBI non-treated **(C,D)** and to a lesser degree in FMT treated **(E,F)** animals. FMT treatment led to a significantly reduced lesion volume **(G)** and lesioned percent of the ipsilateral hemisphere **(H)** at 7 days as compared to 1 day post TBI. TBI non-treated animals experienced a significant change in MLS as measured at the falx cerebri **(I)** and at the septum pellucidum **(J)**, while FMT treated animals showed no changes in MLS. All data is expressed as mean ± SEM. Bar denotes a time effect within treatment. **p < 0.01 and *p < 0.05.

To evaluate the effect of FMT (fecal microbiota transplant abbreviation; Table 1) treatment on structural TBI pathology in the brain, FSE T2W MRI sequences were evaluated at 1 and 7 days post TBI (Figures 1A–F). Lesion volume was significantly decreased in FMT treated animals at 7 days post injury as compared to 1 day post (6.041 ± 1.233 vs. 11.089 ± 1.233 cm^3^, respectively, *p* = 0.001; Figure 1G), while non-treated TBI control animal lesion volumes did not differ between day 7 and day 1 (6.918 ± 1.233 vs. 9.636 ± 1.233 cm^3^, respectively, *p* = 0.079; Figure 1G). Nominal lesion injuries were detected in Sham animals due to craniectomy surgery and lesions did not change at 7 days as compared to 1 day post-surgery (0.021 ± 1.233 vs. 0.062 ± 1.233 cm^3^, respectively, *p* = 1.000; Figure 1G).

**TABLE 1:** Frequently used abbreviations.

Abbreviation	Meaning
TBI	Traumatic brain injury
GIT	Gastrointestinal tract
MGBA	Microbiota-gut-brain-axis
HPA	Hypothalamic-pituitary-adrenal
FMT	Fecal microbial transplant
CCI	Controlled cortical impact
FSE T2W	Fast Spin Echo T2-Weighted
SWAN	Susceptibility-weighted angiography
DWI	Diffusion weighted imaging
MRI	Magnetic resonance imaging
MRS	Magnetic resonance spectroscopy
MLS	Midline shift
ADC	Apparent diffusion coefficient
NAA	N-acetyl aspartate
NAAG	N-acetyl aspartyl glutamate
Cr	Creatine
PCr	Phosphocreatine
Lac	Lactate
Glu	Glutamate
GFAP	Glial fibrillary acidic protein
DCX	Doublecortin

In order to account for possible differences in brain size, the percent of ipsilateral tissue occupied by the lesion was calculated for all groups. The percent of ipsilateral brain containing lesion tissue was significantly less in FMT pigs at 7 days as compared to 1 day post TBI (12.299% ± 2.327% vs. 21.727% ± 2.327%, respectively, *p* = 0.020; Figure 1H). No significant changes were observed from 7 to 1 day in TBI non-treated control (14.255% ± 2.327% vs. 18.908% ± 2.327%, respectively, *p* = 0.118; Figure 1H). Additionally, Sham animals did not exhibit a change in lesion tissue from 7 days as compared to 1 day (0.041% ± 2.327% vs. 0.124% ± 2.327%, respectively, *p* = 1.000; Figure 1H) post TBI. This data suggests FMT contributed to a reduction in lesion volume following TBI.

Midline shift severity is directly correlated with poor clinical outcomes making it an important injury/recovery biomarker (Palekar et al., 2021). MLS was calculated at 1 and 7 days post injury utilizing the falx cerebri, as the injury was more dorsal, as well as the more commonly utilized septum pellucidum. The midline significantly shifted between 1 and 7 days post injury in the non-treated TBI group (0.233 ± 0.443 vs. −1.858 ± 0.44 mm, respectively, *p* < 0.001; Figure 1I) measured at the falx cerebri and at the level of the septum pellucidum (−0.082 ± 0.388 vs. −1.417 ± 0.388 mm, respectively, *p* = 0.044; Figure 1J). There was no significant difference in MLS from 1 to 7 days post injury in the FMT treated group at the falx cerebri (0.483 ± 0.443 vs. −0.525 ± 0.443 mm, respectively, *p* = 0.111; Figure 1I) or the septum pellucidum (0.475 ± 0.388 vs. −0.650 ± 0.388 mm, respectively, *p* = 0.112; Figure 1J). The Sham group demonstrated no change between the 1 and 7 day timepoints at the falx cerebri (0.008 ± 0.443 vs. −0.033 ± 0.443 mm, respectively, *p* = 1.00; Figure 1I) nor the septum pellucidum (−0.008 ± 0.388 vs. 0.008 ± 0.388 mm, respectively, *p* = 1.00; Figure 1J). Negative values demonstrate a shift toward the ipsilateral hemisphere, indicative of tissue atrophy. Therefore, our MLS data suggest FMT-treated pigs may have experienced less tissue atrophy 7 days post injury.

### FMT reduces intracranial hemorrhage volume

**FIGURE 2:**
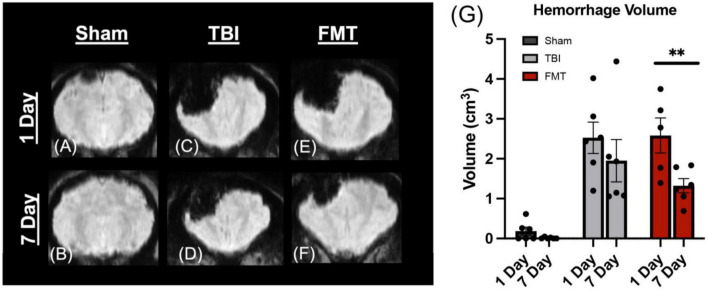
FMT significantly reduced hemorrhage volume in TBI piglets. Coronal SWAN images showed hypointense regions indicative of intracerebral blood and blood products **(A–F)**. Sham animals show minimal hemorrhage at 1 day **(A)** and 7 days **(B)**. TBI non-treated animals showed a slight decrease in hypointense hemorrhagic lesion volume from 1 day **(C)** to 7 days **(D)** post TBI whereas FMT treated animals showed a significant reduction in hypointense hemorrhagic lesion volume from 1 day **(E)** to 7 days **(F)** post TBI **(G)**. Data is presented as mean ± SEM. **p < 0.01.

The SWAN MRI sequence is a sensitive measure of cerebral hemorrhage (Docampo et al., 2013). Others have reported a reduction in hemorrhagic brain injury as a result of microbiota transplant (Yu et al., 2021), therefore we investigated the effects of FMT on hemorrhage volume 1 and 7 days post injury (Figures 2A–F). A decrease in hemorrhagic volume was noted in FMT treated pigs at 7 days as compared to 1 day post injury (1.323 ± 0.321 vs. 2.543 ± 0.337 cm^3^, respectively; *p* = 0.003; Figure 2G). A significant decrease was not observed in non-treated TBI pigs at 7 days as compared to 1 day post injury (1.950 ± 0.321 vs. 2.523 ± 0.321 cm^3^, respectively; *p* = 0.212 Figure 2G). As expected, Sham animals showed minimal cerebral hemorrhage at 1 day and 7 day (0.186 ± 0.321 cm^3^ vs. 0.012 ± 0.321 cm^3^, respectively; *p* = 0.975; Figure 2G). These results indicate FMT therapy may alleviate intracranial hemorrhage associated with moderate-severe TBI.

### FMT reduces cytotoxic and vasogenic edema

**FIGURE 3:**
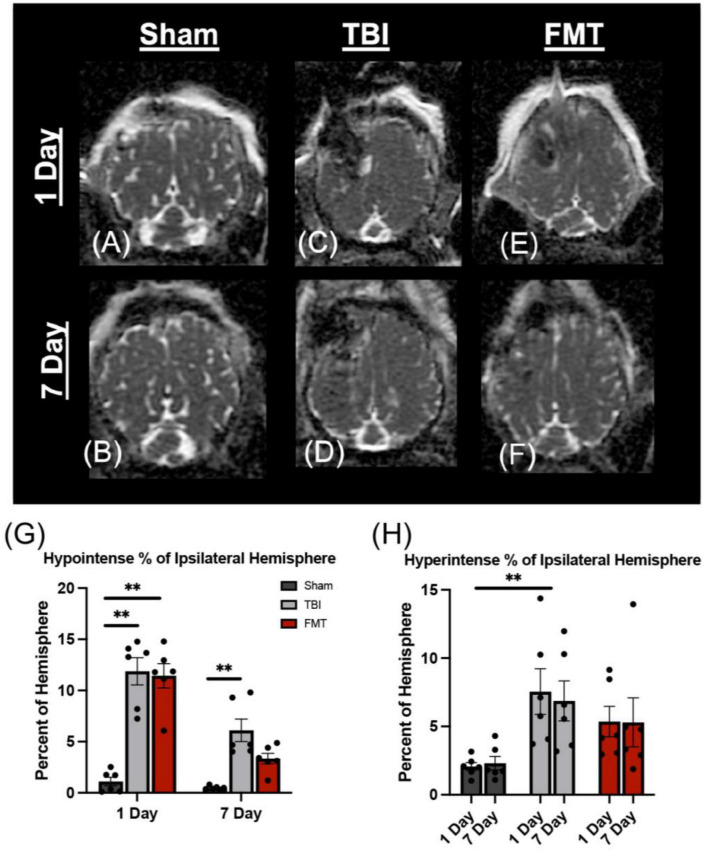
FMT ameliorates cytotoxic and vasogenic edema. Axial ADC maps showed minimal edema at 1 day **(A)** and 7 days **(B)** in Sham animals. However, the percentage of the ipsilateral hemisphere occupied by cytotoxic edema (hypointense regions) was significantly elevated in TBI non-treated **(C)** and FMT treated **(E)** animals at 1 day post TBI as compared to Sham **(A,G)**. However, the percentage of the ipsilateral hemisphere occupied by cytotoxic edema was not significantly different between Sham **(B)** and FMT **(F)** treated animals at 7 days post TBI, while cytotoxic edema remained significantly elevated in TBI non-treated animals **(D,G)**. An overall treatment effect was reported in the percent of the ipsilateral hemisphere occupied by vasogenic edema (hyperintense regions) in which TBI non-treated animals **(C,D)** displayed a significantly greater area of hyperintensity whereas there was no difference in vasogenic edema in FMT treated animals **(E,F)** compared to Sham animals **(A,B,H)**. Data is presented as mean ± SEM. Treatment effect within day is compared to Sham. Bracket indicates treatment difference compared to Sham. **p < 0.01.

Apparent diffusion coefficient maps generated from DWI MRI sequences were used to evaluate changes in water diffusivity in the ipsilateral hemisphere (Figures 3A–F). TBI causes a heterogeneous injury that includes hypointense and hyperintense regions of cytotoxic and vasogenic edema, respectively. Therefore, ipsilateral hemisphere analysis was used to identify and quantitate changes in hypointense and hyperintense regions. At 1 day post TBI, as compared to Sham (1.100 ± 0.912%), both FMT treated (11.436 ± 0.912%, *p* < 0.001) and TBI non-treated (11.998 ± 0.912%, *p* < 0.001) animals had significantly increased levels of hypointensity (Figure 3G). At 7 days post TBI, a decrease was observed in which FMT treated pigs did not differ from Sham (3.343 ± 0.912 vs. 0.474 ± 0.912%, respectively, *p* = 0.283), but non-treated TBI pigs hypointense regions remained increased as compared to Sham (6.104 ± 0.912% vs. 0.474 ± 0.912%, respectively, *p* = 0.006; Figure 3G). An overall treatment difference was observed for hyperintensity (*p* < 0.001), indicative of vasogenic edema in the ipsilateral hemisphere. TBI non-treated animals (7.549 ± 1.077%) showed a significant (*p* = 0.004) increase in hyperintensity relative to Sham (2.069 ± 1.077%; Figure 3H). FMT treated animals (5.358 ± 1.077%) showed no difference as compared to Sham (*p* = 0.070) or TBI non-treated animals (*p* = 0.341; Figure 3H). Together these results indicate administration of FMT may lead to the reduction of cytotoxic and vasogenic edema at 7 days post TBI.

### FMT alters key brain metabolite levels

Magnetic resonance spectroscopy enables non-invasive, quantitative evaluation of brain metabolites, which have been reported to be altered after TBI (Croall et al., 2015; Kinder et al., 2019b). Commonly measured metabolites include N-acetylaspartate (NAA; neuron death and dysfunction), N-acetyl aspartyl glutamic acid (NAAG; membrane turnover), NAA and NAAG (NAA + NAAG), lactate (Lac; glycolysis), glutamate (Glu; neuronal death), phosphocreatine (PCr), creatine (Cr), and the sum of creatine and phosphocreatine (Cr + PCr; cellular energy). Although no treatment by time interactions were observed, a significant treatment effect was noted for multiple metabolites. The NAAG concentration was significantly affected by treatment (*p* = 0.021) as TBI non-treated animals exhibited lower NAAG concentration as compared to Sham (1.080 ± 0.209 vs. 1.974 ± 0.195 IU, respectively, *p* = 0.019) but FMT treated pigs did not statistically differ from Sham (1.739 ± 0.199 vs. 1.974 ± 0.195 IU *p* = 0.682; Supplementary Figure 1A), which may indicate a neuroprotective effect (Neale et al., 2011). A significant decrease was observed in PCr (*p* = 0.022) in TBI non-treated pigs compared to Sham (4.503 ± 0.490 vs. 6.411 ± 0.457 IU, *p* = 0.017), but FMT treated pigs did not statistically differ from Sham (5.376 ± 0.468 vs. 6.411 ± 0.457 IU, respectively, *p* = 0.262; Supplementary Figure 1B), suggesting FMT promoted proper energy metabolism in the TBI brain. A significant treatment difference was also detected in NAA + NAAG concentration (*p* = 0.013) in which TBI non-treated (14.279 ± 0.476 IU, *p* = 0.017) and FMT treated (14.629 ± 0.454 IU, *p* = 0.042) animals were lower relative to Sham (16.339 ± 0.446 IU; Supplementary Figure 1C). No difference was reported for Lac between TBI non-treated, Sham, and FMT treated animals (0.489 ± 0.258 vs. 1.271 ± 0.241 vs. 0.787 ± 0.246 IU, respectively, *p* = 0.097; Supplementary Figure 1D). A significant treatment effect was noted for NAA (*p* = 0.043) in which the concentration of FMT treated animals was significantly decreased as compared to Sham (14.369 ± 0.442 vs. 16.009 ± 0.434, respectively, *p* = 0.046), but TBI non-treated animals did not significantly differ from Sham (14.707 ± 0.464 vs. 16.009 ± 0.434, respectively, *p* = 0.135; Supplementary Figure 1E). The concentration of Cr + PCr approached a trending (*p* = 0.097) treatment difference, but *post-hoc* analysis revealed no statistical differences between the three groups (*p* > 0.100; Supplementary Figure 1F). No statistically significant differences between groups were noted for Glu (*p* = 0.672; Supplementary Figure 1G) or Cr (*p* = 0.534; Supplementary Figure 1H). These data suggest FMT treatment may contribute to moderate recovery of brain metabolism following TBI.

### FMT improved neuron and oligodendrocyte survival

**FIGURE 4:**
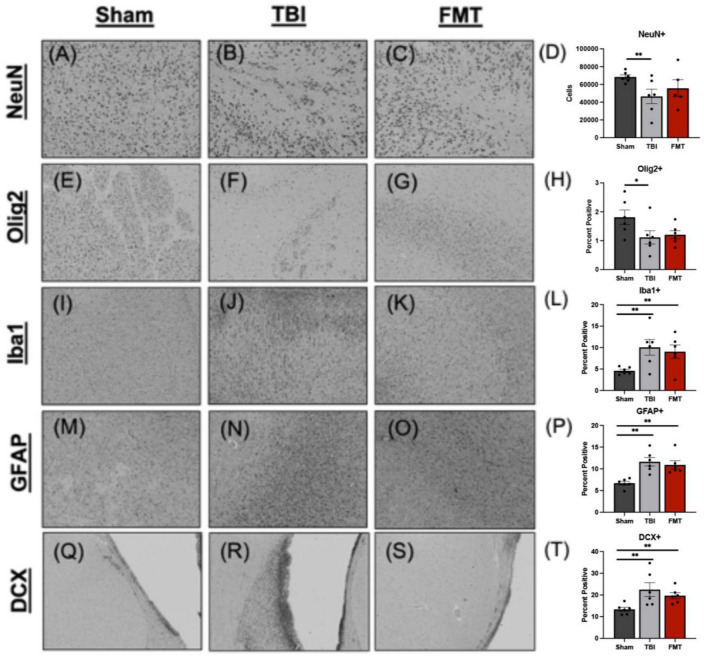
FMT increases neuron survival in TBI animals at 7 days post TBI. Histological evaluation of NeuN+ neurons in Sham **(A)**, TBI non-treated **(B)**, and FMT treated animals **(C)** showed TBI non-treated animals demonstrated a significant decrease in NeuN+ neurons relative to Sham animals, while FMT treated animals **(C)** showed no difference from Sham **(D)**. Histological assessment of Olig2+ oligodendrocytes in Sham **(E)**, TBI non-treated **(F)**, and FMT treated animals **(G)** showed TBI non-treated animals demonstrated a significant decrease in Olig2+ relative to Sham animals, while FMT treated animals did not **(H)**. Iba1+ activated microglia in Sham **(I)**, TBI non-treated **(J)**, and FMT treated **(K)** animals showed TBI non-treated and FMT treated animals displayed a significant increase in Iba1+ microglia cells as compared to Sham animals **(L)**. GFAP+ astrocytes in Sham **(M)**, TBI non-treated **(N)**, and FMT treated **(O)** animals demonstrated TBI non-treated and FMT treated animals showed a significant increase in GFAP+ astrocyte activation as compared to Sham animals **(P)**. DCX+ neuroblasts in Sham **(Q)**, TBI non-treated **(R)**, and FMT treated **(S)** animals demonstrated TBI non-treated animals showed a significant increase in DCX+ cells as compared to FMT treated and Sham animals **(T)**. Additionally, FMT treated animals had a significant increase in DCX+ cells as compared to Sham **(T)**. All images were captured at 10 × magnification. Data is presented as mean ± SEM. Treatment effects are as compared to Sham. **p < 0.01 and * *p* < 0.05.

Ipsilateral tissue sections underwent histological assessment to determine cellular level changes in neuron survival, gliosis, microglia and astrocyte activation, and neuroblast stimulation in response to FMT treatment (Supplementary Figure 2). There was a difference between treatments in number of surviving NeuN+ neurons (*p* = 0.007). A decrease was observed in the number of surviving NeuN+ neurons in TBI non-treated animals (*p* = 0.005) but not in FMT treated animals (*p* = 0.181), as compared to Sham (45133.000 ± 4121.000 and 53990.000 ± 4514.000 vs. 65090.000 ± 4121.000 cells, respectively; Figures 4A–D), indicating less neuronal death in FMT treated animals compared to TBI non-treated. Similarly, the percent area positive for Olig2+ oligodendrocytes was different (*p* = 0.018) between treatments. There was a decrease in the percentage of area positive for Olig2+ oligodendrocytes in TBI non-treated animals (*p* = 0.013) but not in FMT treated animals (*p* = 0.214) as compared to Sham (1.126 ± 0.134 and 1.371 ± 0.134 vs. 1.697 ± 0.134%, respectively; Figures 4E–H), demonstrating improved oligodendrocyte survival in FMT treated animals compared to TBI-non-treated. TBI induced an increase in the percent of Iba1+ microglia activated (*p* < 0.001) in TBI non-treated animals and FMT treated animals as compared to Sham (9.779 ± 0.864 and 8.526 ± 0.864 vs. 4.339 ± 0.864%, respectively, *p* < 0.005; Figures 4I–L). This data demonstrates TBI led to an increase in the percent of activated microglia thus increasing harmful neural inflammation. FMT did not decrease astrocyte activation as TBI non-treated and FMT-treated animals both demonstrated significantly increased GFAP + percent area as compared to Sham (11.859 ± 0.655 and 11.397 ± 0.655 vs. 6.441 ± 0.655%, respectively; (*p* < 0.001); Figures 4M–P). However, there was not a difference in GFAP + percent area between TBI non-treated and FMT treated animals (11.859 ± 0.655 vs. 11.397 ± 0.655%, respectively; (*p* = 0.622); Figures 4M–P) demonstrating FMT treatment did not influence subacute astrocyte activation. Finally, the number of DCX + neuroblasts was evaluated at the subventricular zone. TBI non-treated and FMT treated animals showed a significant increase in the percent area of DCX + neuroblasts as compared Sham animals (15.555 ± 0.859 vs. 13.377 ± 0.859 and 8.003 ± 0.859%, *p* ≤ 0.004; Figures 4Q–T). It should be noted that changes Olig2 + oligodendrocytes, Iba1 + microglia, GFAP + astrocytes, and DCX + neuroblast markers may be due to an increase in cell number or a change in morphology as staining was quantified on a per pixel basis. Collectively, these data indicate that FMT treatment improves neuron survival and has limited effects on other cellular responses in TBI animals.

### FMT did not alter alpha diversity or phylum level in feces

No changes in fecal sample alpha diversity indices were detected as measured by number of features, Faith’s Phylogenetic Diversity (PD) index, Shannon index, and Evenness index in 1, 3, and 7 day post TBI fecal samples (Supplementary Table 1) or 7 day post TBI cecal samples (Supplementary Table 2). The microbial composition of donor FMT treatment (Supplementary Figure 3A) contained 20 different phyla at relative abundances of 63.2% Bacillota (formerly Firmicutes), 30.5% Bacteroidota, 2.5% Spirochaetota, 2.4% Actinobacteriota, and 0.7% Pseudomonadota (formerly Proteobacteria) with all other phyla comprising less than 0.5% of the total relative abundance. The overall composition of day 7 cecal bacterial phyla can be found in Supplementary Figure 3B for Sham, FMT, and TBI piglets and Supplementary Figure 3C for individual piglets. The overall composition of fecal bacterial phyla can be found in Supplementary Figure 3D for Sham, FMT, and TBI piglets pre-TBI and 1, 3, and 7 days post TBI and Supplementary Figure 3E for individual piglets pre-TBI and 1, 3, and 7 days post TBI. Visual differences were observed between phyla; however, to draw more meaningful conclusions, further analyses of the gut microbiome were focused on bacterial species.

### FMT prevented pathogenic bacterial increase in feces

**FIGURE 5:**
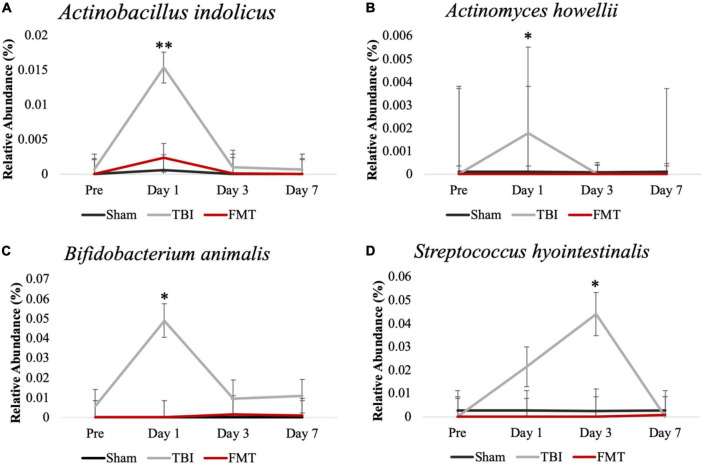
FMT prevented pathogenic bacterial increase in feces of FMT treated piglets at acute timepoints. Fecal microbiome analysis of *Actinobacillus indolicus* **(A)**, *Actinomyces howellii* **(B)**, *Bifidobacterium animalis* **(C)**, and *Streptococcus hyointestinalis* **(D)** showed that TBI non-treated animals had an acute increase in potentially pathogenic bacteria compared to FMT treated and Sham animals. Data is presented as mean ± SEM. **p < 0.01 and *p < 0.05.

Fecal bacterial species relative abundances (Figure 5) were measured via 16S rRNA sequencing to detect acute changes in GIT microbiota post TBI and measure effects of FMT treatment on these populations. *Actinobacillus indolicus* (Figure 5A), a known pathogenic bacterium, made up a relatively small portion of the microbial population of Sham, TBI and FMT piglets prior to surgery (0.000 ± 0.002 0.001 ± 0.002 and 0.000 ± 0.002, respectively). However, there was a significant increase (0.015 ± 0.002; *p* = 0.007) in the relative abundance of *A. indolicus* 1 day post TBI in TBI non-treated piglets compared to FMT treated and Sham piglets (0.001 ± 0.002 and 0.002 ± 0.002, respectively). Fecal relative abundances of *Actinomyces howellii* (Figure 5B) was increased (0.002 ± 0.004) 1 day post TBI in TBI non-treated piglets compared to Sham and FMT treated piglets (0.000 ± 0.004 and 0.000 ± 0.004, respectively; *p* = 0.043). Similarly, *Bifidobacterium animalis* (Figure 5C) had a trending increase in relative abundance 1 day post TBI in TBI non-treated piglets compared to FMT treated and Sham piglets (00.048 ± 0.0085 vs. 0.000 ± 0.0085 and 0.000 ± 0.0085, respectively; *p* = 0.067). Fecal relative abundances of *Streptococcus hyointestinalis* (Figure 5D) increased 3 days post TBI in TBI-non treated piglets compared to FMT treated and Sham piglets (0.044 ± 0.008 vs. 0 ± 0.008 and 0.003 ± 0.008, respectively; *p* = 0.020). Collectively, these data indicated TBI resulted in an acute increase in harmful bacteria which can be prevented by FMT treatment.

### FMT increased probiotic cecal bacteria

**FIGURE 6:**
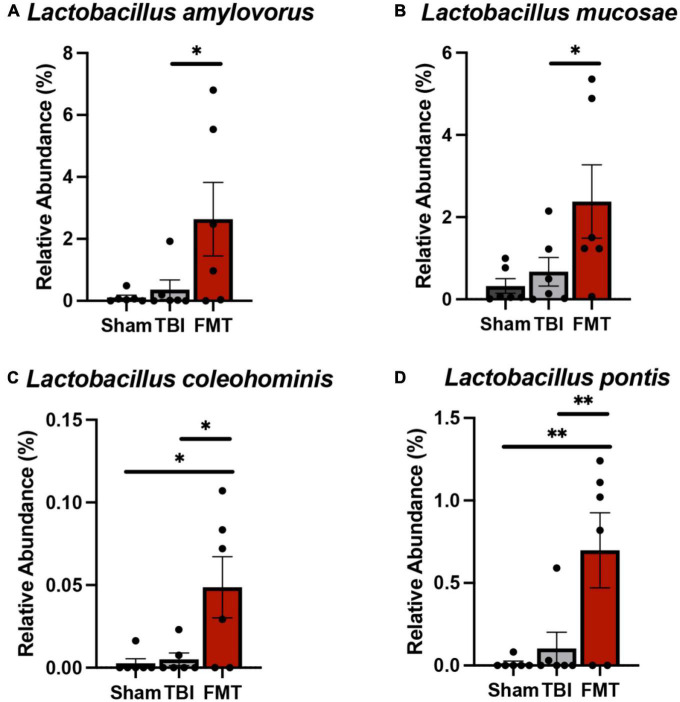
FMT increased probiotic cecal bacteria at 7 days post TBI. Cecal microbiome analysis revealed significant increases in probiotic species *L. amylovorus* **(A)** and *L. mucosae* **(B)** in the cecum of FMT treated animals versus TBI non-treated animals. *L. coleohominis* **(C)** and *L. pontis* **(D)** were increased in FMT treated animals compared to TBI non-treated and Sham animals. Data is presented as mean ± SEM. **p < 0.01 and *p < 0.05.

Cecal relative abundances of bacterial species (Figure 6) were measured 7 days post TBI to determine the effects of FMT on intestinal microbial populations. A number of differences were measured in cecal relative abundances of beneficial bacteria. The relative abundance of *L. amylovorus* (Figure 6A) and *L. mucosae* (Figure 6B) was increased in FMT treated piglets compared to TBI non-treated piglets (2.637 ± 0.784 vs. 0.971 ± 0.784; *p* = 0.024 and 2.38 ± 0.607 vs. 0.000 ± 0.607; *p* = 0.023, respectively). *Lactobacilli coleohominis* (Figure 6C) and *L. pontis* (Figure 6D) was increased in cecal contents of FMT treated piglets compared to TBI non-treated and Sham piglets (0.049 ± 0.014 vs. 0.006 ± 0.014 and 0.002 ± 0.014; *p* = 0.027 and 0.698 ± 0.159 vs. 0.173 ± 0.159 and 0.000 ± 0.159; *p* = 0.006; respectively). Collectively, these results indicated FMT treatment may be capable of shifting specific species in gut populations to an increased proportion of potentially beneficial bacteria, thus preventing gut dysbiosis.

### FMT restores gut villi morphology

**FIGURE 7:**
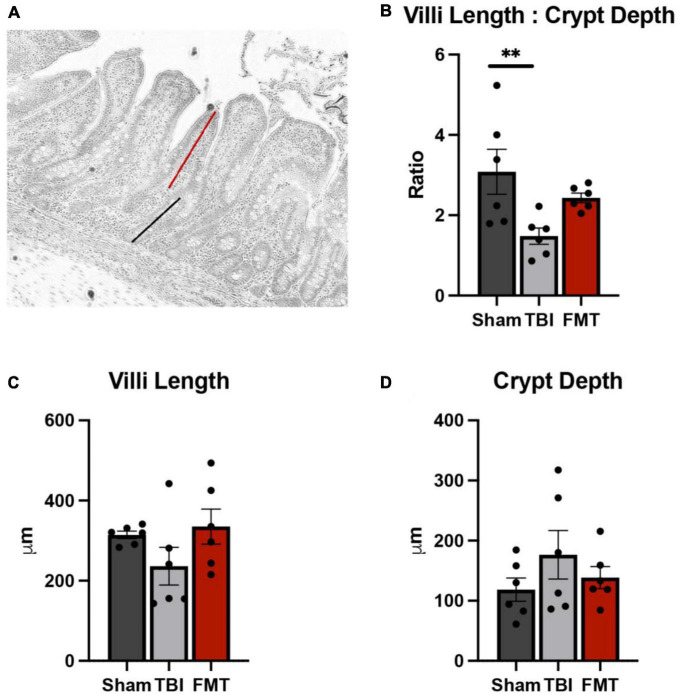
FMT treatment restores gut villi morphology. At 7 days post TBI, ileum tissue was acquired, and morphological measurements including villi height (red line), **(A)** and crypt depth (black line), **(A)** were obtained on 15 intact villi. TBI non-treated animals showed a significant reduction in villi length to crypt depth ratio, while FMT treated animals showed no significant difference **(B)**. No differences were noted between any treatment group in villi length **(C)** and crypt depth **(D)**. Data is presented as mean ± SEM. Treatment effects are as compared to Sham. **p < 0.01.

Ileal tissues were collected and analyzed for changes in villi morphology 7 days post TBI (Figure 7A). A significant difference was observed in TBI non-treated animals in villi length to crypt depth ratio (*p* = 0.018). No differences were noted between Sham and FMT treated animals (3.083 ± 0.351 vs. 2.493 ± 0.351μm, respectively, *p* = 0.412), but TBI non-treated animals (1.480 ± 0.351 μm) demonstrated a significantly reduced ratio as compared to Sham (*p* = 0.015; Figure 7B). However, there was no treatment differences detected for villi length (*p* = 0.180; Figure 7C) between Sham (314.400 ± 37.500 μm), TBI non-treated (236.300 ± 37.500 μm), FMT (335.000 ± 37.500 μm) animals. Additionally, no treatment differences were noted (*p* = 0.351) for crypt depth of TBI non-treated (113.800 ± 13.300 μm, *p* = 0.330) or FMT treated (109.500 ± 13.300 μm, *p* = 0.869) animals as compared to Sham (124.500 ± 713.300 μm) (Figure 7D). Together, these findings suggest FMT administration contributes to an improvement of gut villi morphology ratios.

### FMT promotes motor function recovery

**FIGURE 8:**
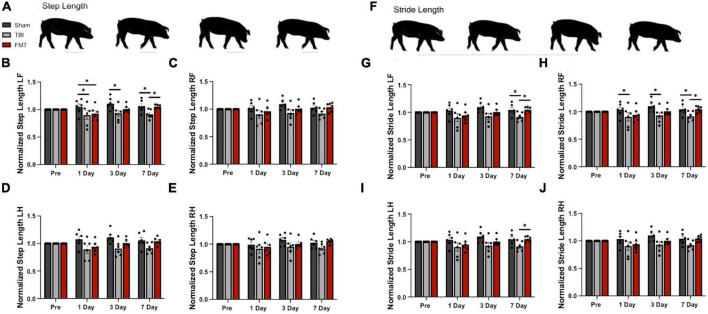
FMT improves step and stride length following TBI. Step length is the measured distance between heel strikes of opposite hooves **(A)**. At 1 day post TBI a decrease in left front step length was noted in TBI non-treated and FMT treated animals as compared to Sham **(B)**. At 3 days post TBI, TBI non-treated animals displayed a decreased step length in the left front **(B)** limbs as compared to Sham. At 7 days post TBI, TBI non-treated animals displayed a decreased step length in the left front **(B)** limbs as compared to Sham and FMT treated animals. However, there was no difference in right front **(C)**, right hind **(E)** or left hind **(D)** step length between treatments. Stride length measures the distance between consecutive heel strikes of the same limb **(F)**. At 1 day post TBI, TBI non-treated pigs exhibited a reduced stride length in the right front **(H)** limb as compared to Sham. At 3 days post TBI, TBI non-treated pigs exhibited a reduced stride length in the left front **(G)** and right front **(H)** limb as compared to Sham. At 7 days post TBI, as compared to Sham and FMT treated animals, TBI non-treated animals showed a decreased stride length in the left front **(G)** and right front **(H)** limbs. At 7 days post TBI, TBI non-treated animals showed a decreased stride length in the left hind **(I)** limbs as compared to FMT treated animals. However, there was no difference in right hind stride length between treatments **(J)**. All data is presented as mean ± SEM. Treatment effects are as compared to Sham. *p < 0.05.

Step length and stride length were normalized to pre-TBI values and analyzed 1, 3, and 7 days post TBI as markers of gait deficits and FMT-mediated recovery. Step length (Figure 8A) is defined as the distance between corresponding contact of opposing hooves, with decreased step length indicating instability. There was a difference in left front step length detected between treatment groups over time (*p* = 0.038; Figure 8B). However, there was no difference in right front (*p* = 0.131; Figure 8C), right hind (*p* = 0.052; Figure 8E), or left hind (*p* = 0.103; Figure 8D) step length between treatments. At 1 day post TBI, TBI non-treated and FMT treated pigs demonstrated a reduction in step length in the left front (0.901 ± 0.042 and 0.927 ± 0.042 vs. 1.045 ± 0.042, *p* ≤ 0.048; Figure 8B) as compared to Sham animals. At 3 days post TBI, TBI non-treated pigs demonstrated a reduction in step length in the left front (0.931 ± 0.042 vs. 1.106 ± 0.042, *p* = 0.005; Figure 8B) as compared to Sham animals, while FMT (1.009 ± 0.042 vs. 1.106 ± 0.042, *p* = 0.005; Figure 8B) did not. At 7 days post TBI, TBI non-treated pigs demonstrated a reduction in step length in the left front (0.916 ± 0.042 vs. 1.060 ± 0.042 and 1.054 ± 0.042, *p* ≤ 0.024; Figure 8B) as compared to Sham and FMT treated animals.

Stride length (Figure 8F) measures the distance between consecutive contacts of the same hoof with a decrease often reported after TBI indicating reduced stability (Neumann et al., 2009; Peri et al., 2019). There was a trending difference in right front (*p* = 0.068; Figure 8H), left front (*p* = 0.067; Figure 8G), and left hind (*p* = 0.085; Figure 8I) stride length between treatment groups. However, there was no difference in right hind stride length between treatments (*p* = 0.102; Figure 8J). At 1 day post TBI, TBI non-treated animals exhibited a reduction in stride length as compared to Sham animals in the right front (0.902 ± 0.041 vs. 1.034 ± 0.041, *p* = 0.029; Figure 8G) limb, but FMT treated animals did not statistically differ from Sham in any limb. At 3 days post TBI, TBI non-treated animals exhibited a reduction in stride length as compared to Sham animals in the left front (0.930 ± 0.041 vs. 1.097 ± 0.041, *p* = 0.029; Figure 8G) and right front (0.933 ± 0.042 vs. 1.100 ± 0.042, *p* = 0.038; Figure 8H), but FMT treated animals did not statistically differ from Sham in any limb. At 7 days post TBI, TBI non-treated animals exhibited a significant reduction in stride length as compared to Sham animals in the left front (0.917 ± 0.041 vs. 1.043 ± 0.041, *p* = 0.035; Figure 8G) and right front (0.918 ± 0.042 vs. 1.045 ± 0.042, *p* = 0.040; Figure 8H). Additionally, the stride length of TBI non-treated animals was decreased as compared to FMT treated animals in the left front (0.917 ± 0.041 vs. 1.045 ± 0.041, *p* = 0.033 Figure 8G), right front (0.918 ± 0.042 vs. 1.048 ± 0.042, *p* = 0.036; Figure 8H) and left hind (0.899 ± 0.037 vs. 1.056 ± 0.045, *p* = 0.014; Figure 8I) limbs. Overall, FMT treated pigs displayed less gait impairment as measured by step length and stride length 3 and 7 days post injury as compared to TBI treated animals, which is indicative of improvement in motor function following TBI.

### FMT improves behavioral deficits caused by TBI

**FIGURE 9:**
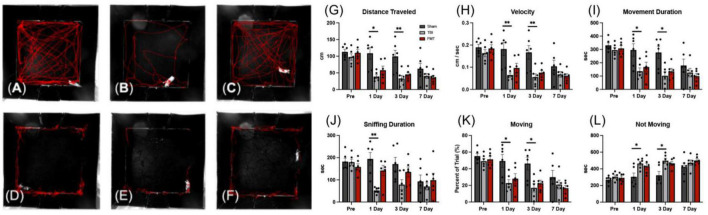
FMT improved voluntary motor activity and exploratory sniffing behavior following TBI. Representative tracking images of distance traveled **(A–C)**; (red lines), and time sniffing **(D–F)**; (red lines) are shown at 1 day post TBI in Sham **(A,D)**, respectively, TBI non treated **(B,E)**, respectively, and FMT treated **(C,F)**, respectively animals. At 1 and 3 days post TBI, TBI non-treated animals traveled significantly less distance **(G)**, moved at a slower velocity **(H)**, spent less time moving during their trial **(I)**, spent a smaller percent of the trial moving **(K)**, and spent more time not moving **(L)** as compared to Sham animals, while FMT treated animals were more comparable to sham. Additionally, at 1 day post TBI, TBI non-treated animals spent less time sniffing **(J)** compared to Sham. Data is presented as mean ± SEM. Treatment effects are as compared to Sham. **p < 0.01 and *p < 0.05.

Traumatic brain injury related behavioral changes were assessed by open field testing pre TBI and 1, 3, and 7 days post TBI. Furthermore, as pigs are inherently exploratory animals, the amount of time an animal spent sniffing the wall of the behavior arena was quantified. There were treatment differences detected overtime for distance traveled (*p* = 0.004; Figures 9A–C), velocity (*p* = 0.004), movement duration (*p* = 0.033), sniff duration (*p* = 0.007; Figures 9D–F), percent of trial moving (*p* = 0.033), and time not moving (*p* = 0.033). In comparing treatments at each timepoint, as expected no treatment differences were observed pre-injury in any measured parameter. At 1 day post TBI, no significant difference was noted between Sham and FMT treated pigs in velocity (0.182 ± 0.019 vs. 0.097 ± 0.019 m/s, respectively, *p* = 0.105; Figure 9H), movement duration (295.980 ± 32.412 s vs. 168.200 ± 32.412, respectively, *p* = 0.220; Figure 9I), sniffing duration (194.193 ± 22.333 vs. 142.127 ± 22.333 s, respectively; *p* = 0.881; Figure 9J), percent of trial moving (49.330 ± 5.402 vs. 28.033 ± 5.402 percent, respectively; *p* = 0.220; Figure 9K), or time not moving (304.020 ± 32.403 vs. 431.800 ± 32.403 s, respectively; *p* = 0.219; Figure 9L). However, at 1 day post TBI, TBI non-treated animals showed a decrease as compared to Sham in velocity (0.065 ± 0.019 vs. 0.182 ± 0.019 m/s, respectively, *p* = 0.004; Figure 9H), movement duration (136.540 ± 32.412 vs. 295.980 ± 32.412 s, respectively, *p* = 0.046; Figure 9I), sniffing duration (52.340 ± 22.333 vs. 194.193 ± 22.333, respectively; *p* = 0.003; Figure 9J), percent of trial moving (22.757 ± 5.402 vs. 49.330 ± 5.402 percent, respectively; *p* = 0.046; Figure 9K). Meanwhile, at 1 day post TBI, TBI non-treated animals had an increase in time not moving compared to Sham (463.460 ± 32.403 vs. 304.020 ± 32.403 s, respectively; *p* = 0.046; Figure 9L). Similarly, 3 days post injury Sham and FMT treated animals only exhibited a trending difference in distance traveled (99.364 ± 11.470 vs. 44.902 ± 11.470, respectively, *p* = 0.062; Figure 9G) and velocity (0.167 ± 0.019 vs. 0.075 ± 0.019, respectively, *p* = 0.059; Figure 9H). Additionally, 3 days post injury Sham and FMT treated animals had no difference in movement duration (277.360 ± 32.412 vs. 135.080 ± 32.412 s, respectively, *p* = 0.113; Figure 9I), percent of trial moving (46.227 ± 5.402 vs. 22.513 ± 5.402 percent, respectively; *p* = 0.113; Figure 9K), or time not moving (322.640 ± 32.403 vs. 464.920 ± 32.403 s, respectively; *p* = 0.113; Figure 9L). However, at 3 days post TBI, TBI non-treated animals showed a decrease relative to Sham in distance traveled (33.322 ± 11.470 vs. 99.364 ± 11.470, respectively, *p* = 0.009; Figure 9G), velocity (0.056 ± 0.019 vs. 0.167 ± 0.019 m/s, respectively, *p* = 0.008; Figure 9H), movement duration (102.053 ± 32.412 vs. 277.360 ± 32.412 s, respectively, *p* = 0.018; Figure 9I), and percent of trial moving (17.009 ± 5.402 vs. 46.227 ± 5.402 percent, respectively; *p* = 0.018; Figure 9K). At 3 days post TBI, TBI non-treated animals had an increase compared to Sham in time spent not moving (497.947 ± 32.403 vs. 464.920 ± 32.403 s, respectively; *p* = 0.018; Figure 9L). There was no significant difference between any of the three treatment groups in behavioral parameters measured 7-days post injury, most likely due to habituation to the behavior arena as has been noted previously (Xiao et al., 2016). Together, this data indicates FMT leads to improved recovery of spontaneous movement and characteristic porcine exploratory behavior.

## Discussion

In this study, we provide, for the first-time, pivotal evidence that FMT can improve cellular and tissue damage, gut microbiota dysbiosis, and functional recovery in a translational pediatric piglet TBI model. Advanced MRI analysis revealed FMT treatment was associated with reduced lesion volume, MLS, hemorrhage volume, and cytotoxic and vasogenic edema. Histological analysis demonstrated that FMT had a neuroprotective leading to increased neuron and oligodendrocyte survival. At the level of the gut, FMT treatment prevented an increase in harmful bacteria, increased beneficial bacterial populations, and restored villi morphology ratios to a pre-TBI like state in treated pigs. Ultimately, these FMT mediated cellular and tissue level improvements were associated with enhanced functional recovery. Gait analysis showed preserved step and stride length and open field testing showed increased voluntary movement including distance traveled, velocity, percent of trial moving, movement duration while decreasing time not moving. Together these findings suggest that daily FMT may be a highly effective therapy for pediatric TBI patients, highlighting the potential of this treatment as a promising avenue for future clinical research.

Magnetic resonance imaging analysis demonstrated significant improvements in TBI-related tissue damage as a result of FMT administration. Lesion volume and MLS, reliable clinical predictors of TBI outcomes in patients (Chastain et al., 2009; Wang et al., 2021), showed FMT administration led to a 45% reduction in lesion volume and a 32 and 54% decrease in MLS at the falx cerebri and septum pellucidum, respectively, at the 7 day timepoint. Similarly, others have reported a reduced lesion volume via *ex vivo* brain tissue analysis at 3 and 7 days post severe CCI as a result of *Lactobacillus acidophils* probiotic administration in a rodent model (Ma et al., 2019). To the best of the author’s knowledge, this is the first study that has reported MLS as a metric of FMT-mediated recovery. However, as a related metric, FMT administration reportedly attenuated cortical volume loss, which is known to be a contributing factor to MLS, at 3 days post severe CCI injury as compared to TBI-injured mice (Davis et al., 2022).

Intracerebral hemorrhage occurs in approximately 40% of children with TBI and are directly related to increased morbidity and mortality in pediatric TBI patients (Tong et al., 2004; Beauchamp et al., 2013). Hemorrhagic transformation has been shown to be closely associated with gut microbiome perturbations (Huang et al., 2022). The present study showed, for the first time, FMT of healthy bacterial populations induced a 48% decrease in TBI hemorrhage volume along with functional recovery.

Apparent diffusion coefficient maps allow for quantification of cytotoxic (restricted diffusion) and vasogenic (increased diffusion) edema (Ashwal et al., 2006) which constitutes excessive intra- and extra-cellular water in the brain (Michinaga and Koyama, 2015). Such fluid accumulation has been reported to begin earlier, to be detected more frequently, and is associated with worst outcomes and higher risk for mortality following pediatric TBI as opposed to adult (Tong et al., 2004; Ichkova et al., 2017). This study found FMT treated pigs exhibited a greater reduction in cytotoxic and vasogenic edema (70% vs. 50% reduction, respectively) as compared to TBI non-treated animals, potentially due to restoration of the blood brain barrier (BBB) (Galea, 2021). This data is in agreement with other preclinical studies that demonstrated probiotic administration decreased edema and improved BBB functionality as compared to non-treated TBI rodents at 24 h and 3 days after TBI (Li et al., 2018; Ma et al., 2019). Together, our clinically relevant, multisequence MRI evaluation approach revealed that FMT led to significant improvements in brain tissue following TBI.

Neural injury has been well documented by our group and others to induce gastrointestinal distress including a shift in microbial population, with dysbiosis correlated to increased lesion volume in porcine and rodent neural injury models (Nicholson et al., 2019; Jeon et al., 2020). Our study demonstrated the ability of FMT to restore specific populations of gut bacterial species after dysbiosis occurred post TBI including reducing acute increases of bacteria that can have harmful effects on the host while increasing probiotic *Lactobacilli* species. Currently, research focusing on specific bacterial species impact on the gut-brain axis is limited. Most research has focused on bacterial phylum level differences. However, with the diverse array of bacterial species within a phylum, including those that are pathogenic and therapeutic, drawing conclusions at higher taxonomic levels can be misleading. Many of the bacterial species that acutely increased in TBI non-treated piglets post TBI have previously been reported to have potentially harmful effects on the host (Jeon et al., 2022). Previously, *A. indolicus* populations were decreased in the gut of piglets receiving colistin, an anti-inflammatory feed additive used to promote animal growth and health (Ali et al., 2022), suggesting this bacterium is associated with an inflamed GIT tract. Similarly, *A. howellii* has been associated with the disease actinomycosis (Ali et al., 2022) in humans which is indicative of this bacteria’s pathogenic nature suggesting the gut of TBI non-treated piglets in the current study were in a dysbiotic diseased state. Similarly, administration of *B. animalis* subsp. *Animalis* previously caused duodenal inflammation and mild colitis in gnotobiotic mice (Moran et al., 2009) and increased in abundance in gnotobiotic mice that have colitis compared to mice without colitis (Bibiloni et al., 2005). Based on previous research on *B. animalis*’ effects on inflammation in mice, TBI non-treated piglets in the current study could have had an increase in the relative abundance of *B. animalis* subsp. *Animalis* due to its associated with inflammation and GIT distress. However, further research is needed to confirm the bacterial subspecies. Finally, increases in *S. hyointestinalis* observed in TBI non-treated piglets at 3 days post TBI may be indicative of gut dysbiosis that has systemic impacts on host health because this bacterium has previously been correlated to peripheral (Duarte and Kim, 2022) and neuroinflammation (Zhou et al., 2020) as well as depressive-like behaviors in mice (Tian et al., 2019). Collectively, our results indicate that FMT treatment may inhibit the growth of potentially harmful bacteria that are associated with disease, inflammation, and GIT distress.

*Lactobacillus*, a bacterial genus from the most abundant phylum Bacillota, has been identified as an important probiotic for gut health due to its anti-inflammatory effects. Specific species such as *L. acidophilus* show promising neuroprotective effects and the ability to restore gut dysbiosis and intestinal smooth muscle contractility post TBI (Masel and DeWitt, 2010; Davis et al., 2022). The present study identified four *Lactobacilli, L. amylovorus, L. coleohominis, L. mucosae*, and *L. pontis*, that were increased in cecal relative abundances in FMT piglets, suggesting their gut populations were less dysbiotic and may have a neuroprotective effect. *Lactobacilli amylovorus* has previously been shown to decrease inflammation by preventing activation of toll-like receptor 4 (TLR4) signaling pathways (Finamore et al., 2014) while also inhibiting growth of many intestinal porcine pathogens *in vitro* (Hynönen et al., 2014). *Lactobacilli coleohominis*, which increased in abundance in the gut microbiota of piglets overtime, produces antimicrobial metabolites (Hu et al., 2016) that can prevent dysbiosis and improve intestinal health. *Lactobacilli mucosae* has been identified to reduce acute inflammation in piglets following a lipopolysaccharide (LPS) challenge by increasing mucosal production of immunoglobulin A (Li et al., 2021). Additionally, it has been shown to alleviate neuropsychiatric disorders in mice by modulating their gut microbiota (Kim et al., 2020) by preventing GIT pathogen colonization and subsequent LPS production that causes GIT and hippocampal inflammation. *Lactobacilli pontis*, which was increased in the cecum of FMT-treated piglets in the current study, was recently isolated from the gut of pigs after probiotic administration. However, this bacterium’s role in the gut microbiota and its effects on immunity has not been fully elucidated (Satora et al., 2020). Our results are indicative of the ability of FMT to increase the relative abundance of many *Lactobacilli* species, thus improving the health of the gut and ultimately the host.

Recovery of gross and fine motor skills and motor coordination is often stated as a patient’s primary goal of TBI rehabilitation, yet severe TBI frequently results in long term gait impairment in children despite significant rehabilitation efforts (Katz-Leurer et al., 2008; Prasad et al., 2017). Our study found TBI induced a significant decrease in step and stride length, consistent with gait changes in piglet (Baker et al., 2019; Kinder et al., 2019b; Sneed et al., 2020) and human (Kuhtz-Buschbeck et al., 2003; Katz-Leurer et al., 2008) pediatric TBI populations. Additionally, we observed decreases in open field voluntary motor parameters including distance traveled time spent moving and exploratory behaviors, such as sniffing, following TBI. In the present study, FMT treatment led to significant gait improvements as well as in voluntary movement and exploratory behavior measures. To date, no rodent studies have conducted gait analysis following FMT or probiotic therapy for TBI. However, rodent studies of gut microbial therapeutics following TBI have reported beneficial functional outcomes including reduced anxiety and improved neurologic function as measured by neurological severity score (Li et al., 2018; Ma et al., 2019; Du et al., 2021), rotarod performance (Ma et al., 2019), Morris Water Maze, (Du et al., 2021), and Elevated Zero Maze (Davis et al., 2022). Maintained exploratory behavior assessed by open field testing was noted here and by Davis et al. (2022) following FMT administration. Therefore, based on the improvement in gait and voluntary movement tests observed herein, FMT treatment shows promise for promoting clinically relevant functional recovery in TBI patients.

Fecal microbial transplant treatment led to significant recovery in TBI animals, which is likely mediated through the MGBA. However, questions remain as to which MGBA component mediates the FMT therapeutic effect- stimulation of the enteric nervous system, activation of the hypothalamic pituitary adrenal axis, dampening systemic inflammation, or potentially all 3 arms of the MGBA axis. The authors hypothesize that FMT administration may contribute to improvement following TBI via anti-inflammatory mediators. Inflammation is a key consideration in the progression of TBI, as early inflammation is important to clear dead cells and debris, however, extended inflammation can exasperate brain damage (Simon et al., 2017). As demonstrated by the current study and others, brain damage can result in changes to the gut (Crapser et al., 2016; Fung et al., 2017; Nicholson et al., 2019). This can lead to an increased systemic inflammatory response, which may further exacerbate neural damage. Increased levels of systemic cytokines have been detected in serum over a year following TBI (Kumar et al., 2015). At the level of the gut, probiotic bacteria have been shown to interact with toll-like receptors to stimulate the immune system, thus altering levels of circulating cytokines (Kashiwagi et al., 2015; Maranduba et al., 2015). In rodent TBI models, administration of *Lactobacillus acidophilus (L. acidophilus)* or *Clostridium butyricum* (*C. butyricum*) inhibited the inflammatory response leading to alterations in the gut microbiome and correlating brain and functional recovery (Li et al., 2018; Ma et al., 2019). In a mouse TBI weight drop model, animals treated with *L. acidophilus* showed higher levels of GIT tight junction protein occludin and prevention of inflammatory leaky gut syndrome as indicated by reduced circulating endotoxin levels (Ma et al., 2019). This resulted in decreased circulating TNF-α levels and brain inflammatory activity including microglia proliferation and *Tlr4* and *Myd88* gene expression. Changes in neuroinflammation were associated with a reduction in BBB damage and edema and ∼50% reduction in lesion volume. These cellular and tissue level changes led to significant improvements in neurological severity scores (NSS) and motor function. Similarly, treatment with *C. butyricum* in a mouse TBI weight drop model decreased IL-6 levels in the colon and increased occludin levels, thus indicating decreased GI inflammation and improved integrity GIT integrity (Li et al., 2018). *Clostridium butyricum* treatment also correlated with a significant reduction in BBB damage, edema, cortical apoptosis, and neurodegeneration relative to controls. These results again correlated with a significant improvement in NSS scores. However, further studies are needed to confirm this effect in our porcine model. While positive results were noted in our all-male cohort, sex differences in response to pediatric TBI have been reported including increased neuroinflammation in males relative to females, likely due to differences in circulating hormones (Arambula et al., 2019). Furthermore, age and sex have been shown to alter the components of the gut microbiome (Yoon and Kim, 2021). Therefore, possible sex differences in response to FMT following TBI should be investigated in future studies.

The therapeutic effect of FMT in TBI animals are expected to persist even after discontinuation of the FMT treatment as the microbial bacteria population can restore the dysbioitic GIT microbiota. The increase in probiotic bacteria in the cecum of FMT treated animals demonstrates the ability of the FMT to reestablish beneficial bacteria in the microbiota. Further studies are needed to confirm the longevity of the FMT TBI therapeutic effect. Beyond FMT treatment, modulation of the gut microbiome to limit TBI injury and promote recovery can potentially be accomplished through other approaches such as consumption of bioactive foods or probiotic supplementation. Fermented foods such as yogurt, apple cider vinegar, cheese, kimchi, kombucha, miso, and sauerkraut (NIH, 2023) contain live microorganisms that may provide a similar probiotic effect to the GIT microbiota as a FMT. However, further research needs to be conducted to determine if adding fermented foods to the diet could have a therapeutic effect following a TBI.

## Conclusion

For the first time in a highly translatable pediatric piglet TBI model, our study has demonstrated the potential of FMT treatment to significantly limit tissue damage, prevent gut dysbiosis, and ultimately improve functional outcomes following TBI. Treatment with FMT resulted in significant therapeutic efficacy as assessed by neuro- and gastrointestinal-histology, advanced neuroimaging, microbiome analysis, and gait and behavior functional testing. Our study highlights the strong therapeutic potential of FMT treatment for TBI and underscores the need for further investigations into its long-term effects and mechanistic pathways. Such studies will be critical in establishing the efficacy and safety of FMT to be evaluated for potential use in human clinical trials.

## Data availability statement

The datasets presented in this study can be found in online repositories. The names of the repository/repositories and accession number(s) can be found below: https://figshare.com/, 10.6084/m9.figshare.23681328.

## Ethics statement

The animal study was approved by the University of Georgia Institutional Animal Care and Use Committee (Animal Use Protocol A2019 07-007-Y1-A9). The study was conducted in accordance with the local legislation and institutional requirements.

## Author contributions

MF, CW, KD, and FW contributed to the conception and design of the study. MF, CW, KS, SS, JJ, MG, and SC performed the pig work. MF, CW, KS, SS, JJ, DB, GO, QZ, and JL performed the data analysis. MF and CW wrote the first draft of the manuscript. All authors contributed to manuscript revision, read, and approved the submitted version.

## Supplementary material

Supplementary Figure 1 FMT improves brain metabolites post TBI. As compared to Sham, TBI non-treated animals exhibited a significant decrease in NAAG **(A)** and PCr **(B)**, while FMT treated pigs did not. TBI non-treated and FMT treated pigs displayed a significant reduction in NAA + NAAG as compared to Sham **(C)**. FMT treated animals showed a significant decrease in NAA as compared to Sham animals **(E)**. No treatment differences were reported in Lac, **(D)**, Cr + PCr **(F)**, Glu **(G)**, or Cr **(H)**. Data is presented as mean ± SEM. Treatment effects are as compared to Sham. ***p* < 0.01 and * *p* < 0.05.

Supplementary Figure 2 A consistent location was used for histological imaging of neural cells. Following euthanasia, brains were immediately removed and placed in a custom piglet brain slicer to ensure consistency in brain sectioning. The rostral portion of the brain (black box) was placed in 10% buffered formalin for immunohistochemical analysis. After fixation, the brain was further subdivided (red box) and tissues were routinely processed, paraffin embedded, sectioned, and serial sections were stained.

Supplementary Figure 3 FMT caused visual changes at the phylum level post TBI. Microbiome analysis of FMT treatment administered to FMT treated piglets **(A)**. Microbiome analysis of the cecal [average per treatment: **(B)**; individual piglets (*n* = 6/treatment)]: **(C)** and fecal [average per treatment: **(D)**; individual piglets (*n* = 6/treatment pre-TBI and 1 and 7 days post TBI; *n* = 5/treatment 3 days post TBI)]: **(E)** environments at the phylum level indicated there were visual differences. There were increased cecal relative abundances of Bacteroidota and Pseudomonadota and decreased cecal relative abundances of Actinobacteriota in TBI non-treated animals 7 days post TBI (B). There were increased fecal relative abundances of Pseudomonadota and Actinobacteriota on 1 day post TBI in TBI non-treated animals and Spirochaetota on day 1 post TBI in FMT treated and TBI non-treated animals **(D)**.
