# Supplementary material for: Fecal microbial transplantation limits neural injury severity and functional deficits in a pediatric piglet traumatic brain injury model
Source: Front Neurosci. 2023 Sep 28;17:1249539. doi: 10.3389/fnins.2023.1249539 (PMC10568032; doi:10.3389/fnins.2023.1249539)
Supplement: Supplementary file 1 [file Table_1.DOCX]

Supplemental Table 1. FMT treatment did not result in alpha diversity differences in the feces.

Supplemental Table 2. FMT treatment did not result in alpha diversity differences in the cecum.
