# Supplementary figures and images for: Fecal microbial transplantation limits neural injury severity and functional deficits in a pediatric piglet traumatic brain injury model
Source: Front Neurosci. 2023 Sep 28;17:1249539. doi: 10.3389/fnins.2023.1249539 (PMC10568032; doi:10.3389/fnins.2023.1249539)

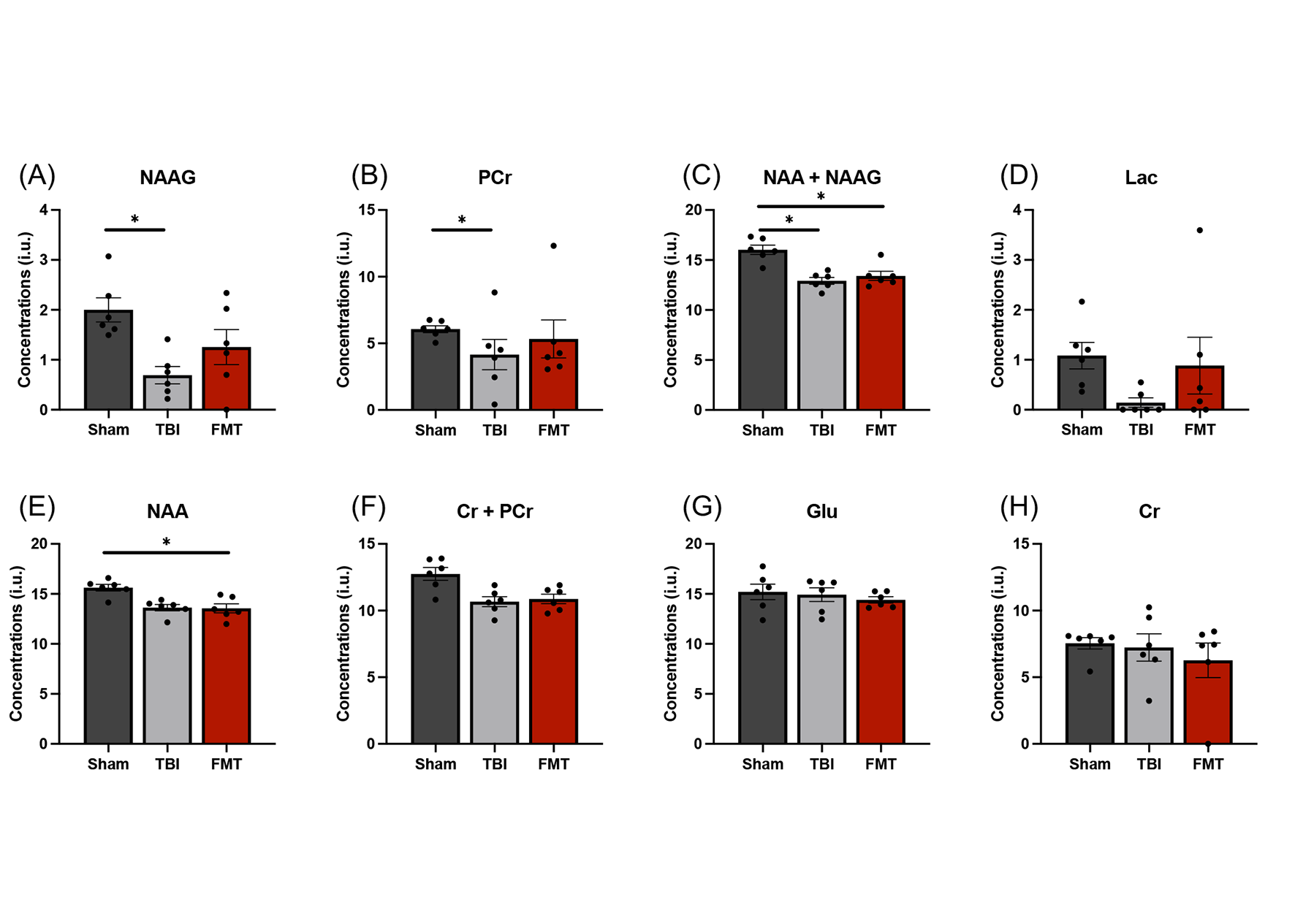

Supplement: Supplementary Figure 1 — FMT improves brain metabolites post TBI. As compared to Sham, TBI non-treated animals exhibited a significant decrease in NAAG (A) and PCr (B), while FMT treated pigs did not. TBI non-treated and FMT treated pigs displayed a significant reduction in NAA + NAAG as compared to Sham (C). FMT treated animals showed a significant decrease in NAA as compared to Sham animals (E). No treatment differences were reported in Lac, (D), Cr + PCr (F), Glu (G), or Cr (H). Data is presented as mean ± SEM. Treatment effects are as compared to Sham. **p < 0.01 and * p < 0.05. [file Image_1.TIF]

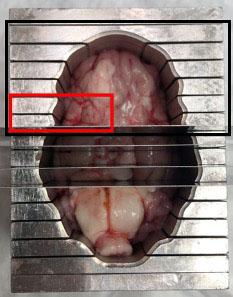

Supplement: Supplementary Figure 2 — A consistent location was used for histological imaging of neural cells. Following euthanasia, brains were immediately removed and placed in a custom piglet brain slicer to ensure consistency in brain sectioning. The rostral portion of the brain (black box) was placed in 10% buffered formalin for immunohistochemical analysis. After fixation, the brain was further subdivided (red box) and tissues were routinely processed, paraffin embedded, sectioned, and serial sections were stained. [file Image_2.JPEG]

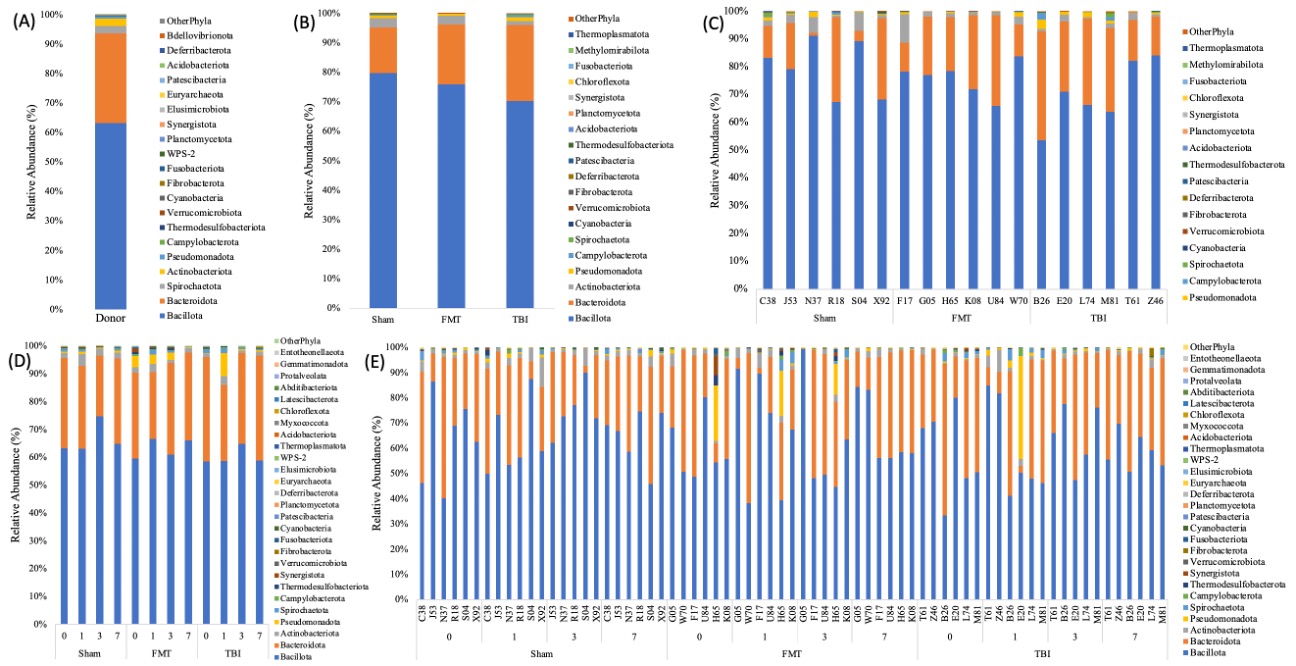

Supplement: Supplementary Figure 3 — FMT caused visual changes at the phylum level post TBI. Microbiome analysis of FMT treatment administered to FMT treated piglets (A). Microbiome analysis of the cecal [average per treatment: (B); individual piglets (n = 6/treatment)]: (C) and fecal [average per treatment: (D); individual piglets (n = 6/treatment pre-TBI and 1 and 7 days post TBI; n = 5/treatment 3 days post TBI)]: (E) environments at the phylum level indicated there were visual differences. There were increased cecal relative abundances of Bacteroidota and Pseudomonadota and decreased cecal relative abundances of Actinobacteriota in TBI non-treated animals 7 days post TBI (B). There were increased fecal relative abundances of Pseudomonadota and Actinobacteriota on 1 day post TBI in TBI non-treated animals and Spirochaetota on day 1 post TBI in FMT treated and TBI non-treated animals (D). [file Image_3.JPEG]
